# A 6D Pose Estimation for Robotic Bin-Picking Using Point-Pair Features with Curvature (Cur-PPF)

**DOI:** 10.3390/s22051805

**Published:** 2022-02-24

**Authors:** Xining Cui, Menghui Yu, Linqigao Wu, Shiqian Wu

**Affiliations:** Institute of Robotics and Intelligent Systems, School of Information Science and Engineering, Wuhan University of Science and Technology, Wuhan 430081, China; cuixining@wust.edu.cn (X.C.); yumenghui_hui@163.com (M.Y.); wulin_a@126.com (L.W.)

**Keywords:** pose estimation, robotic bin-picking, candidate targets, curvature information, weighted voting

## Abstract

Pose estimation is a particularly important link in the task of robotic bin-picking. Its purpose is to obtain the 6D pose (3D position and 3D posture) of the target object. In real bin-picking scenarios, noise, overlap, and occlusion affect accuracy of pose estimation and lead to failure in robot grasping. In this paper, a new point-pair feature (PPF) descriptor is proposed, in which curvature information of point-pairs is introduced to strengthen feature description, and improves the point cloud matching rate. The proposed method also introduces an effective point cloud preprocessing, which extracts candidate targets in complex scenarios, and, thus, improves the overall computational efficiency. By combining with the curvature distribution, a weighted voting scheme is presented to further improve the accuracy of pose estimation. The experimental results performed on public data set and real scenarios show that the accuracy of the proposed method is much higher than that of the existing PPF method, and it is more efficient than the PPF method. The proposed method can be used for robotic bin-picking in real industrial scenarios.

## 1. Introduction

Bin-picking is a common scene in the industry, aiming to take out objects placed in disorder by robotic arms. There are different degrees of overlap and occlusion interference with the detection and perception of objects, yielding the failure of the robotic grasping task [1]. Bin-picking is challenging, attracting many domestic and foreign scholars [2,3,4]. The key of bin-picking is to calculate the pose of the best picking point of the target object [5], namely, 6D pose estimation. According to the current research on pose estimation, it can be divided into correspondence method, template-based method, voting-based method, and deep learning-based method [6].

The method to find the relationship between input data and known point cloud model is called the correspondence method. According to the type of input data, the method can be divided into 2D–3D correspondence and 3D–3D correspondence [7]. The 2D–3D corresponding method is often used for objects with rich textures. The point cloud model is projected from multiple angles, and the relationship between the template image and the RGB image of the target object in a single angle, is found through feature points. Then, the Perspective-n-Point (PnP) algorithm is used to restore the pose of the current perspective. For example, Hu et al. [8] introduced a segmentation driven network framework for 6D pose estimation. This method predicts the local pose through the 2D key point position of objects in the scenario, thereby generating a set of reliable 3D to 2D correspondences, and then uses the PnP algorithm to calculate the accurate pose of each object. This method can maintain robustness in the presence of overlap among objects, but it is not suitable for untextured objects. In the 3D–3D corresponding method, the acquired depth image is converted into a 3D point cloud, and then the relationship between the two point clouds is solved through the point cloud descriptor. The common point cloud descriptors include Fast Point Feature Histogram (FPFH) [9], Signature of Histogram of Orientation (SHOT) [10], Oriented FAST and Rotated BRIEF (ORB) [11], and so on. For example, Xue et al. [12] proposed an improved Iterative Closest Point (ICP) [13] for point cloud registration. In this method, the initial registration point-pairs are selected by FPFH, then Principal Component Analysis (PCA) algorithm is used for coarse registration, and finally, the improved ICP algorithm is used for fine registration. This method takes a long time to load the point cloud, and registration parameters need to be continuously optimized, so that it is not suitable for industrial pipeline tasks.

Template-based methods are usually used for weakly textured or untextured objects, which are difficult to extract feature points. The principle of the method is to select the most similar template with the object in the scene, and regard the template pose as one of the objects. Usually, the template is the complete point cloud of the object, and the pose calculation is formulated as a local registration problem, i.e., align the input single-view point cloud with the complete template point cloud. For example, Sarode et al. [14] proposed a new point cloud registration network (PCRNet). This method is based on the prior information of the point cloud shape, and the transformation matrix is obtained by comparing the global features of the template point cloud with the target point cloud. This method is robust to point cloud noise and the initial deviation of the pose, but is not suitable for occlusion scenarios. Hence, this method cannot be used for bin-picking.

Voting-based methods are based on each part of target objects to be able to vote on the overall output [15]. Local voting usually refers to the vote of each pixel or 3D point to obtain the final 6D poses of target objects. Such methods are often used in scenarios where there are no texture and overlapping occlusions among objects, which are suitable for robotic arms to perform bin-picking tasks. Methods based on voting strategies can be divided into indirect voting and direct voting. The former is to obtain a predefined feature point by voting for each pixel or 3D point, and obtain a 6D pose according to the 2D–3D or 3D–3D correspondence. For example, Peng et al. [16] proposed to use Pixel-wise Voting Network (PVNet) to return unit vectors to key points, then used RANdom SAmple Consensus (RANSAC) to vote for key points, and finally used PnP algorithm to derive accurate poses. This method relies on the key points of the 2D target object, and is not applicable to objects which are self-similar without texture information. Direct voting is to directly obtain a certain 6D pose by voting between each pixel or 3D point. For example, Drost et al. [17] defined a new four-dimensional point-pair feature to describe the object. Through feature matching, the relationship between scene point-pairs and model point-pairs is modeled. The generated candidate poses are voted to obtain the final result. This method performs well in scenes with noise, clutter, and partial occlusion, and, accordingly, is suitable for complex industrial environments.

In recent years, deep learning has made great breakthroughs in artificial intelligence [18]. Applying this method to robotic arms can improve the applicability of grasping. For example, Wang et al. [19] proposed a new dense fusion network to obtain pixel-level dense feature, thereby obtaining the pose of the target object. The advantage of this method is that an iterative fine-tuning process is integrated into the network architecture, which eliminates the dependence of fine pose on ICP, and is also robust to occlusion situations. But color and depth information are needed by the network, which adds complexity and cost. Braun et al. [20] designed a new method for joint target detection and pose prediction of deep convolutional networks. The disadvantage of this method is that it does not apply overlap and occlusion, and the estimation accuracy of the pose of small objects is not ideal.

As Drost’s method [17] can well cope with complex scenarios, this method has been deeply studied in recent years, to make it play the best effect for different scenarios. For example, Choi et al. [21] improved the method by using color information. Thus, the four-dimensional point-pair feature is formed into a ten-dimensional point-pair feature of point cloud, which greatly improves the matching rate of colored objects. Liu et al. [22] improved the feature description of industrial parts based on the original method. In this method, the normal vector in the original feature is changed to a tangent vector to enhance the feature representation of objects. A multi-edge appearance method of model description was proposed, to improve the efficiency by reducing useless point-pairs matching. Vidal et al. [23] presented to estimate 6D poses of free-form objects in the presence of clutter and occlusions. By considering the judgment value of surface information, a new viewpoint-dependent re-scoring process and two scene consistency verification steps were proposed to reduce false-positive cases. Ruel et al. [24] proposed a 3DLASSO system which was designed to perform real-time tracking and 6D pose estimation of target spacecraft from sparse and noisy 3D data. Different from the PPF method, instead of point-pairs larger polygons are used in a similar setup, and a faster version of the ICP algorithm is developed for pose estimation. The algorithm is quite robust to sensor noise and deviations from the reference model, but poses that do not provide enough geometric information to the algorithm showed larger errors.

In order to solve the bin-picking problem in industry, we have expanded and improved Drost’s method [17]. The main contributions of the proposed method are: (1) An effective method for extracting candidate targets point cloud is adopted in preprocessing step. Specifically, the organized scene point cloud is mapped to the grayscale image, and the segmented grayscale images are mapped back to the point cloud. After threshold processing, only point cloud of unobstructed target objects in the scene are retained; (2) A new point-pair feature descriptor is proposed, which introduces curvature information based on the PPF method to effectively enhance the description of point-pair features; (3) In the pose voting link, a new weighted voting scheme is proposed by combining the curvature distribution of the model, which gives more weight to high information point-pairs, thereby further improving the accuracy of pose estimation.

The rest of this paper is organized as follows. The proposed method is presented in Section 2. Experimental results and discussions are given in Section 3. The conclusion is provided in Section 4.

## 2. The Proposed Method

**Figure 1:**
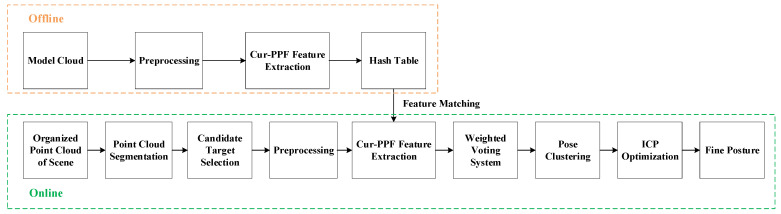
The full point-pair feature with curvature pipeline. The proposed method can be divided into offline stage and online stage.

Our work is based on the method proposed by Drost et al. [17]. Through the improvement and optimization of the PPF, 6D poses of target objects can be accurately achieved in complex industrial scenarios, enabling the robotic arm to complete the bin-picking tasks. The flow chart of the proposed method is shown in Figure 1, which comprises offline phase and online phase.

**Figure 2:**
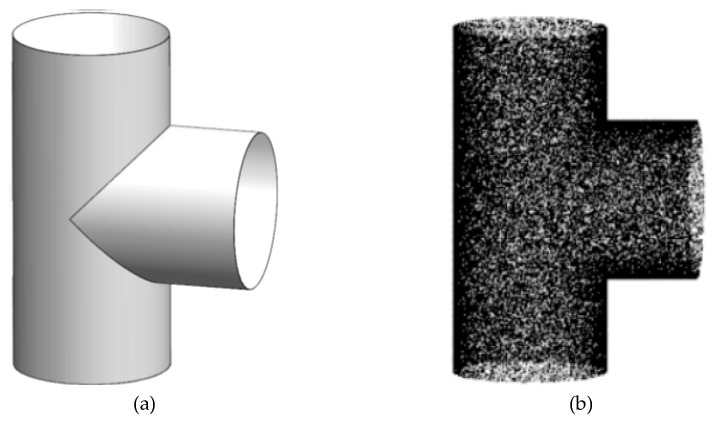
The three-way tube is one of models in our experiments. (**a**) is the CAD model of the three-way tube, and (**b**) is the point cloud model of the three-way tube after sampling.

In the offline stage, the CAD model of the object is used to generate point cloud, as shown in Figure 2. Firstly, the generated model point cloud is preprocessed, which mainly includes point cloud downsampling, normal calculation, and curvature calculation. Due to the mass of model point cloud, it causes calculation redundancy. In order to speed up the processing, downsampling operation is required. The normals and curvatures of point cloud are calculated to prepare for PPF [25]. Then the high-curvature part and the low-curvature part are classified according to curvature distributions of models point cloud, and the pose weighted voting is performed on point-pairs with high information. Finally, the six-dimensional features of the model point-pairs are calculated, and features are stored in the hash table for features matching in the online phase.

In the online stage, the main work is to calculate 6D poses of target objects through PPF matching, to achieve precise grasping. We use a 3D sensor to obtain organized scene point cloud, which is outlier removed and transformed into gray images through mapping. Watershed algorithm is used [26] to segment gray images and candidate targets are extracted. For the segmented point cloud, the same preprocessing and features calculation are performed as done in the offline stage. By finding PPFs similar to target objects in the hash table, transformations among model point-pairs and scene point-pairs are derived, and the weighted votes of poses are completed in the two-dimensional accumulator. Finally, poses are clustered and the average of the highest clustered poses is used as the output result. The ICP algorithm is used to refine the pose estimation. In the next section, we will elaborate on all aspects of the proposed method, especially the differences from the PPF.

### 2.1. Offline Phase

#### 2.1.1. Preprocessing

The preprocessing includes point cloud downsampling, normal calculation and curvature calculation. The point cloud downsampling and normal calculation are the same as the method by Drost et al. [17]. In the following, we focus on the point cloud curvature calculation.

Curvature can reflect the bending degree of geometry [27]. In the three-dimensional space, the curvature of the point cloud can provide special information for feature matching, which can effectively reduce matching error [28]. From the geometric description, the types of curvature can be divided into principal curvature, Gaussian curvature and average curvature. Principal curvature refers to the normal curvature in the principal direction of a point on the surface, and it is also the maximum and minimum values of the normal curvature of the surface in all directions at that point. At any point in the point cloud, there is a surface z=r(x,y) approaching this point. Assuming that the principal curvature of this point is $k_{n}$, the $k_{n}$ calculation formula is:

(1) $$\left|\begin{array}{cc} L-k_{n}E & M-k_{n}F\\ M-k_{N}F & N-k_{n}G \end{array}\right|=0,$$

(2) $$\left(EG-F^{2}\right)k_{n}^{2}-\left(LG-2MF+NE\right)k_{n}+\left(LN-M^{2}\right)=0.$$

The principal curvature $k_{n}$ is obtained by solving the quadratic equation. In the formula $E=r_{x}r_{x},F=r_{x}r_{y},G=r_{y}r_{y},L=r_{xx}n,M=r_{xy}n,N=r_{yy}n$; where $r_{x},r_{y},r_{xx},r_{yy},r_{xy}$ is the partial differential of the surface z=r(x,y), n is the value of the unit normal vector of the tangent plane of the surface z=r(x,y) at the point $\left(x_{0},y_{0}\right)$, that is, $n=\left(r_{x}\times r_{y}\right)/\left|r_{x}\times r_{y}\right||{}_{\left(x_{0},y_{0}\right)}$. (E,F,G) is called the first basic invariant of the surface, and (L,M,N) is called the second basic invariant of the surface. The Gaussian curvature of a point on the surface is the product of the two principal curvatures, which is used to characterize the overall curvature of the local area, denoted as K, that is, $K=k_{1}k_{2}$. The average curvature of a point on the surface is the average of the two principal curvatures, denoted as H, that is, $H=\left(k_{1}+k_{2}\right)/2$. Combining the principal curvature calculation Formula (2) and the Veda theorem, it can be known that the calculation formulas of Gaussian curvature and average curvature are:

(3) $$K=\frac{LN-M^{2}}{EG-F^{2}},$$

(4) $$H=\frac{LG-2MF+NE}{2\left(EG-F^{2}\right)}.$$

In order to better describe the change of the point cloud, we used the average curvature to represent curvature characteristics.

#### 2.1.2. Cur-PPF Feature Extraction and Hash Table

**Figure 3:**
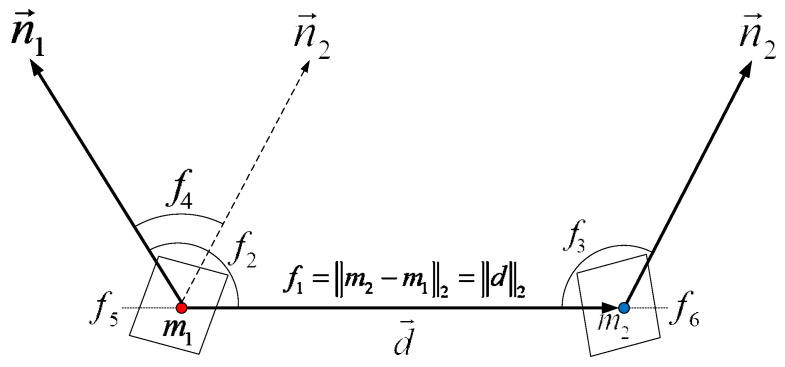
The model description of the Cur-PPF feature. This feature is different from the PPF because curvature information $f_{5}$ and $f_{6}$ are introduced to Cur-PPF, which strengthens the feature description.

**Figure 4:**
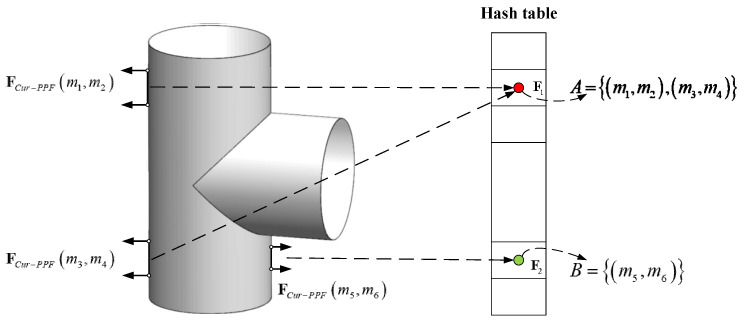
The hash table model. For the three point-pairs on the model, because point-pairs $\left(m_{1},m_{2}\right)$ and $\left(m_{3},m_{4}\right)$ have the same feature description, these two point-pairs are stored in the same slot of the hash table, and the key of the slot is represented by the feature $F_{1}$ of these two point-pairs; the feature description of point $\left(m_{5},m_{6}\right)$ is different from $F_{1}$, so it is stored in another slot of the hash table, which is represented by feature $F_{2}$.

The proposed Cur-PPF is a six-dimensional feature vector using the distance information of two points and its normal vector and average curvature. Compared with the original PPF, curvature information is introduced in the proposed method, which enhances the feature description of point-pairs. Cur-PPF is shown in Figure 3. For any point-pair $\left(m_{1},m_{2}\right)$, $m_{1}$ and $m_{2}$ are two points in the model point cloud, $n_{1}$ and $n_{2}$ are the normal vectors of these two points, $q_{1}$ and $q_{2}$ are average curvatures of the two points, vector $d=m_{2}-m_{1}$, feature expression F is:

(5) $$\begin{array}{l} F_{Cur-PPF}\left(m_{1},m_{2}\right)=\left(f_{1},f_{2},f_{3},f_{4},f_{5},f_{6}\right)\\ =\left({\left\|d\right\|}_{2},∠\left(n_{1},d\right),∠\left(n_{2},d\right),∠\left(n_{1},n_{2}\right),q_{1},q_{2}\right), \end{array}$$

where ${\left\|d\right\|}_{2}$ represents the Euclidean distance between the two points, ∠(a,b)∈[0,π] denotes the angle between two vectors. It should be noted that the feature $F_{Cur-PPF}$ is asymmetric, just as $F_{Cur-PPF}\left(m_{1},m_{2}\right)$ and $F_{Cur-PPF}\left(m_{2},m_{1}\right)$ are not the same. In the offline stage, the model point cloud is represented with a set of similar features $F_{Cur-PPF}$. Here we set the steps of distance, angle and curvature to $d_{dist}$, $d_{angle}$, and $d_{cur}$. Then point-pairs with similar characteristics are placed in the same slot of the hash table, and the keys of the hash table are characteristics of point-pairs, as shown in Figure 4. The model features $F_{Cur-PPF}\left(m_{i},m_{j}\right)$ can be searched in constant time by using $F_{Cur-PPF}\left(s_{i},s_{j}\right)$ as the key to access the hash table.

### 2.2. Online Phase

#### 2.2.1. Point Cloud Segmentation and Candidate Target Selection

Effectively extracting target objects in complex scenarios is very helpful for feature matching, so scene point cloud segmentation is performed. Point cloud segmentation can be divided into two categories [29]. The first type of method is the direct method, in which the point cloud is directly segmented, such as the Euclidean distance segmentation algorithm [30] integrated in the PCL library [31]. Its principle is to find a certain point in space, the n points closest to the point are found through KdTree, and the distance to the point is judged. If the distance is less than the threshold, it is considered to be of the same kind. This algorithm has to traverse all the points in the space, which is complicated and takes a long time, so it is not suitable for real-time system. The second is the indirect method. The point cloud is mapped to a two-dimensional image for segmentation, and then segmented images are mapped back to the three-dimensional space to achieve point cloud segmentation. The method is based on two-dimensional image processing, with high accuracy and less time consumed [32].

Because the point cloud is obtained by the 3D sensor in this system and the order of the point cloud is known [33], we chose the second method to achieve point cloud segmentation. Firstly, the ordered point cloud is projected onto the plane composed of x−axis and y−axis of the coordinate system, and the effective detection range of the depth value in the z−axis direction is mapped to become the gray value. Then the watershed segmentation algorithm [26] is used to segment the gray image, so an image is divided into several disjoint local areas. Finally, gray images are mapped back to the three-dimensional space to complete the point cloud segmentation. For a more detailed understanding of the segmentation process, we describe it using pseudocode, which is shown in Algorithm 1.

**Figure 5:**
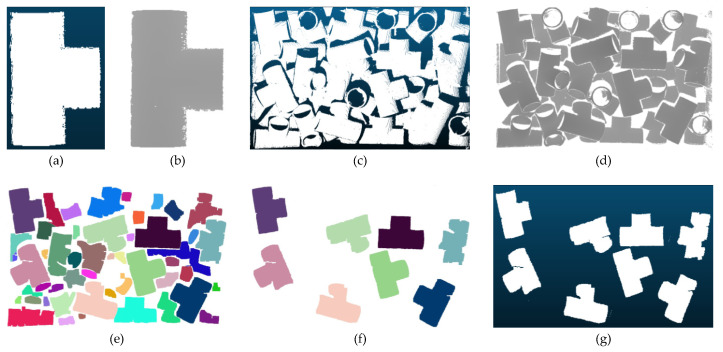
The process of point cloud segmentation and candidate targets selection. (**a**) is the point cloud of the one-sided model; (**b**) is the grayscale image of the one-sided model; (**c**) is the point cloud of the scene; (**d**) is the grayscale image which is mapped from the depth information of the scene; (**e**) is the grayscale image of the scene after segmentation; (**f**) is the grayscale image of the scene after target selecting; and (**g**) is the point cloud which is mapped by (**f**).

There are usually overlapping occlusions in the picking scenarios. The candidate objects grabbed by the robotic arm are the top priority (that is, the ones that are not occluded or have a large exposed surface), which also conforms to the logical order of grabbing. Therefore, grayscale images are thresholded after watershed segmentation. Firstly, the single-sided point cloud of a single object in the scene is obtained by a 3D sensor and mapped to a grayscale image to obtain the number of pixels of the image. Then, the number of local pixels after segmentation are compared with the number of pixels on one side of the object. If the number of surface pixels is similar to the number of surface pixels on one side of the object, and the number of contour pixels is similar to the number of contour pixels on one side of the object, we consider the object to be a candidate to be grasped by the robotic arm. Finally, each pixel is mapped to three-dimensional space to complete the effective segmentation and the selection of candidate targets. Three-way tube is a category in the test data set of this paper, and is demonstrated as a legend, as shown in Figure 5.
**Algorithm 1** Watershed Segmentation Algorithm Based on Distance Transform1: Input: I, Output: O 2: **if** I(i,j)=(255,255,255)    I(i,j)=(0,0,0)    **end if** 3: L←Laplacian operator(I) 4: S←Sharp(L) 5: G←Grayscale(S) 6: **if** $G(i,j)>t_{1}$        G(i,j)=255    **else**        G(i,j)=0    **end if** 7: D←Distance transform(G) 8: N←Normalized(D),N(i,j)⊂[0,1] 9: **if** $N(i,j)>t_{2}$        N(i,j)=255    **else**        N(i,j)=0    **end if** 10: P←Erode(N) 11: M←Find and draw contours(P) 12: O←Watershed(S,M)


#### 2.2.2. Feature Matching

**Figure 6:**
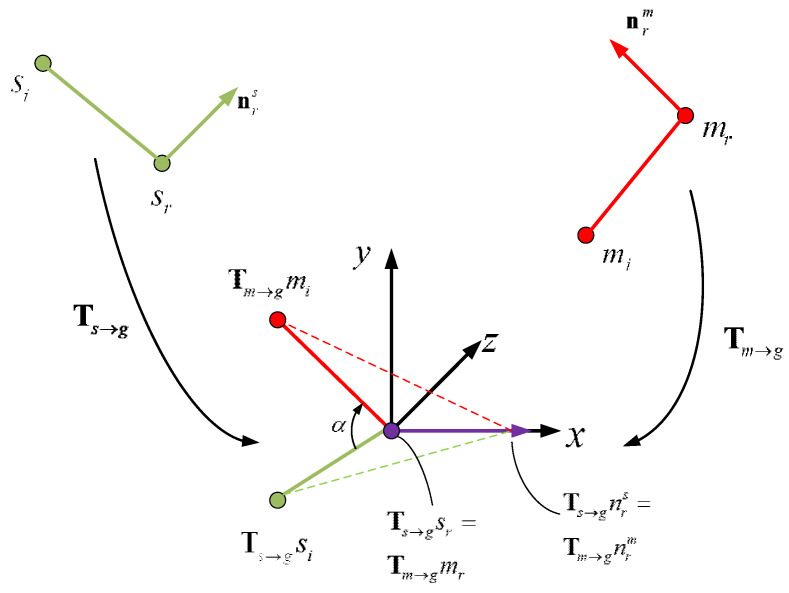
Transformation between model point-pairs and scene point-pairs. The transformation relationship $R_{x}\left(\alpha \right)$ is obtained by aligning the point-pair vector and its normal vector.

Feature matching refers to successfully finding PPFs of the model in the hash table, so that the transformation can be calculated. In this paper, the local coordinate system is established for solving. Given a point-pair $\left(s_{r},s_{j}\right)$ in the scene, the Cur-PPF of the point-pair is calculated and the feature as the key value is used to find the corresponding model point-pair $\left(m_{r},m_{j}\right)$ in the hash table. The two points $s_{r}$ and $m_{r}$ are moved to the origin of the local coordinate system, and the normals of these two points are aligned with the x−axis, so that the object can be rotated around the normal to align the model with the scene, as shown in Figure 6. The transformation from the model to the scene can be represented by a point and a rotation angle α, which is $\left(m_{r},\alpha \right)$. If the model point-pair $\left(m_{r},m_{j}\right)$ and the scene point-pair $\left(s_{r},s_{j}\right)$ have similar Cur-PPF, the conversion relationship between the two point-pairs can be calculated by the Formula (6).

(6) $$s_{i}=T_{s\to g}^{-1}R_{x}\left(\alpha \right)T_{m\to g}m_{i},$$

where, $T_{m\to g}$ is a transformation with rotation and translation, which translates the reference point $m_{r}$ in the model point-pair feature $\left(m_{r},m_{i}\right)$ to the origin of the coordinate system, and at the same time rotates the normal vector $n_{r}^{m}$ of the reference point $m_{r}$ to the same direction as the x−axis of the coordinate system. $T_{s\to g}$ is also a transformation with rotation and translation, which translates the reference point $s_{r}$ in the model point-pair feature $\left(s_{r},s_{i}\right)$ to the origin of the coordinate system, and at the same time rotates the normal vector $n_{r}^{s}$ of the reference point $s_{r}$ to the same direction as the x−axis of the coordinate system. $T_{s\to g}^{-1}$ is the inverse of $T_{s\to g}$. $R_{x}\left(\alpha \right)$ is the rotation around the x−axis with angle α.

In order to improve the calculation speed of α angle, α can be divided into two parts, namely $\alpha =\alpha_{m}-\alpha_{s}$. Where, $\alpha_{m}$ is the rotation angle at which the model point-pair $\left(m_{r},m_{i}\right)$ continues to rotate around the x−axis after the transformation of $T_{m\to g}$, so that the point $m_{i}$ falls on the plane composed of the x−axis and the positive half-axis of the y−axis; $\alpha_{s}$ is the rotation angle at which the scene point-pair $\left(s_{r},s_{i}\right)$ continues to rotate around the x−axis after the transformation of $T_{s\to g}$, so that the point $s_{i}$ falls on the plane composed of the x−axis and the positive half-axis of the y−axis; the direction of rotation of the two remains the same. The calculation of these two parts is independent of each other, so we can split $R_{x}\left(\alpha \right)=R_{x}\left(-\alpha_{s}\right)R_{x}\left(\alpha_{m}\right)$ and use $R_{x}^{-1}\left(-\alpha_{s}\right)=R_{x}\left(\alpha_{s}\right)$ to get

(7) $$t=R_{x}\left(\alpha_{s}\right)T_{s\to g}s_{i}=R_{x}\left(\alpha_{m}\right)T_{m\to g}m_{i}.$$

i.e., t lies on the half-plane defined by the x−axis and the non-negative part of the y−axis. For successfully paired point-pairs, $\alpha_{m}$ can be calculated for model point-pairs in the offline phase and store them in the hash table. In this way, only $\alpha_{s}$ needs to be calculated for scene point-pairs. The final angle α is the difference between the two angles.

#### 2.2.3. Weighted Voting System

We search model point-pairs $\left(m_{r},m_{j}\right)$ with same Cur-PPF features as $\left(s_{r},s_{j}\right)$ from the hash table. Formula (6) is used to calculate mapping relationships α from each model point-pairs $\left(m_{r},m_{j}\right)$ to scene point-pairs $\left(s_{r},s_{j}\right)$. Then we use a method similar to the generalized Hough transform to vote on the obtained α and select the best mapping relationship to restore the global pose of the object.

**Figure 7:**
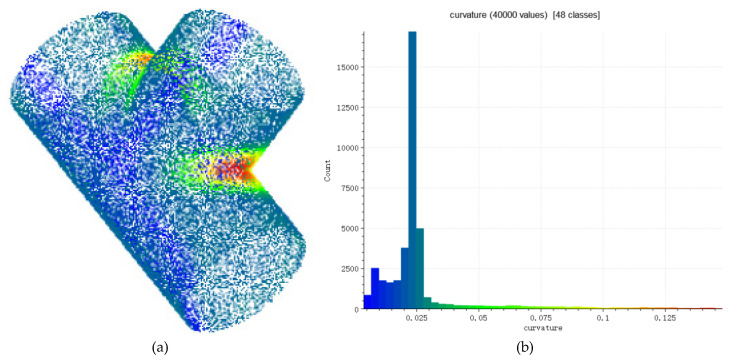
(**a**) is the curvature distribution of the three-way tube model. The color from red to blue corresponds to the average curvature of the point cloud from large to small. (**b**) is the curvature histogram of the three-way tube. According to the curvature histogram, we set 0–0.035 as the low-curvature range, and greater than 0.1 as the high-curvature range.

**Figure 8:**
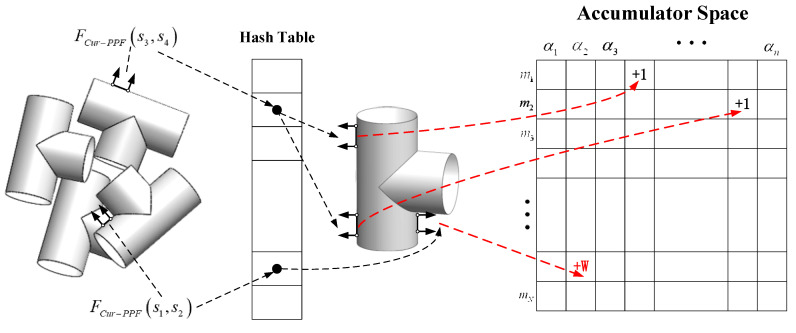
Because the scene point-pair $\left(s_{3},s_{4}\right)$ is matched with two points of the same curvature class in the model point-pair, the number of pose votes is one by default; when matching $\left(s_{1},s_{2}\right)$, the two points in the model point-pair are in the set high-curvature and low-curvature ranges respectively, so the match contains more information and weighted voting is performed on the pose.

Voting process is completed through a two-dimensional accumulator. The rows $N_{m}$ of the accumulator is equivalent to the number of model points M, and the columns $N_{angle}$ is equivalent to the step length $n_{angle}$ of the conversion relationship α. Whenever scene point-pairs $\left(s_{r},s_{j}\right)$ are successfully paired with model point-pairs $\left(m_{r},m_{j}\right)$ in the hash table, the calculated α are voted. The difference from the PPF is that our method combines the model curvature distribution in the actual voting process, and different α votes are assigned different weights. When scene point-pairs and model point-pairs are successfully paired, we will focus on the relationship between the point $m_{r}$ and the point $m_{j}$ in the model point-pair $\left(m_{r},m_{j}\right)$. From Section 2.1.1, the average curvature value of each point in the model point cloud can be calculated. The curvature distribution of the three-way tube model is shown in Figure 7a. Different colors represent the average curvature value. It can be seen from characteristics of curvature that point cloud with similar curvature values is also similar in bending, and such point cloud is distributed in the same area in space. And point cloud with large differences in curvature values also has large differences in the degree of bending, and such point cloud is distributed far apart in space. We believe that point-pairs with the greater difference in curvature values of the two points contain more information, and the mapping relationship α is calculated by the pairing is more accurate, such that α should be given a higher weight when voting, as shown in Formula (8). For example, in the three-way tube model of this experiment, the high-curvature part and low-curvature part of the model are divided according to the curvature histogram. The curvature histogram is shown in Figure 7b. Weighted vote is performed on the calculated α that has a greater difference in curvature values between the two points in the model point-pair. The voting process is shown in Figure 8.

(8) $$\mathrm{Weight}=\{{}_{+1\;other}^{+W\;when\;m_{1}\in high-cur\&m_{2}\in low-cur\;∥\;m_{1}\in low-cur\&m_{2}\in high-cur}.$$

#### 2.2.4. Pose Clustering

When reference points are located on the object surface, multiple effective point-pairs will be generated. Each point-pair will be calculated a pose result after feature matching, so an object will have a set of pose sets. The pose sets are clustered to ensure that the translation and rotation errors of all poses in each category are in the set threshold. The score of each pose is the cumulative sum of votes obtained by that pose during the voting phase. The category with the highest score is selected, and poses contained in this category are averaged to obtain the final pose results. This operation not only removes the pose data with large errors through the threshold, but also improves the accuracy of the final pose result by the average value. Since there will be multiple objects in the scene, multiple high-scoring categories will be generated, and the category with the highest number of votes is selected as the preferred pose.

#### 2.2.5. ICP Optimization

In order to further improve the accuracy of the pose results, we used the ICP algorithm [13] for optimization after the pose obtained by the pose clustering. The clustering pose is used as the initial value of the ICP algorithm, and the error is further reduced by continuously reducing the Euclidean distance between the model point and the corresponding scene point. On basis of whether model points match scenic points successfully by setting the distance threshold. If the distance between the two points is less than the threshold, it is considered that the two points match successfully. Finally, the ratio ∂ between the number of matched points and the number of object points in the scene is taken as the matching rate, as shown in Formula (9). In real experimental scenario, when the value of the matching rate can enable the robotic arm to successfully grasp the target object, it is the minimum matching rate that we can accept.

(9) $$\partial =\frac{\mathrm{Number}\;\mathrm{of}\;\mathrm{matching}\;\mathrm{success}\;\mathrm{points}}{\mathrm{Number}\;\mathrm{of}\;\mathrm{object}\;\mathrm{points}\;\mathrm{in}\;\mathrm{the}\;\mathrm{scene}}.$$

## 3. Experimental Results and Discussions

We used online public data set and real scene data to verify the effectiveness of the proposed method, and used a robotic arm to perform bin-picking tasks to evaluate the performance of the method in industrial applications. Our algorithm was implemented in C++ language under the Visual Studio2019 platform and was run on the NVIDIA GeForce GTX1060 processor. Through experimental comparison, the advantages of the proposed method over the original method are verified in terms of accuracy, efficiency, and adaptability.

### 3.1. Public Data Set

**Figure 9:**
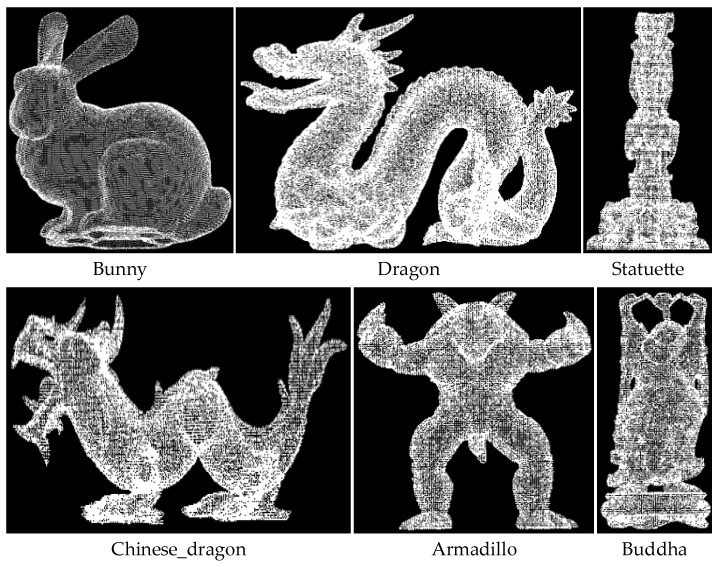
The model point cloud data set. The data set includes six point cloud models, namely, Bunny, Dragon, Statuette, Chinese_Dragon, Armadillo, and Buddha.

We used the online Retrieval [34] data set to verify the advancement of the proposed method. The data set includes 6 models and 18 scenes, and the model is shown in Figure 9. Each scene has only one set of point cloud data, which prevents other factors from interfering with the experimental comparison results. For all experiments, the Leaf_size of the model point cloud and scene point cloud downsampling was set to 5 mm; the hash table distance step $d_{dist}$ was set to 3 mm; the angle step $d_{angle}$ was set to $12^{\circ }$; and the 1/5 of the point cloud number was used as the scene reference point. The matching rate of the point cloud was calculated by Formula (8) in Section 2.2.5, where the threshold was set to 5 mm.

**Figure 10:**
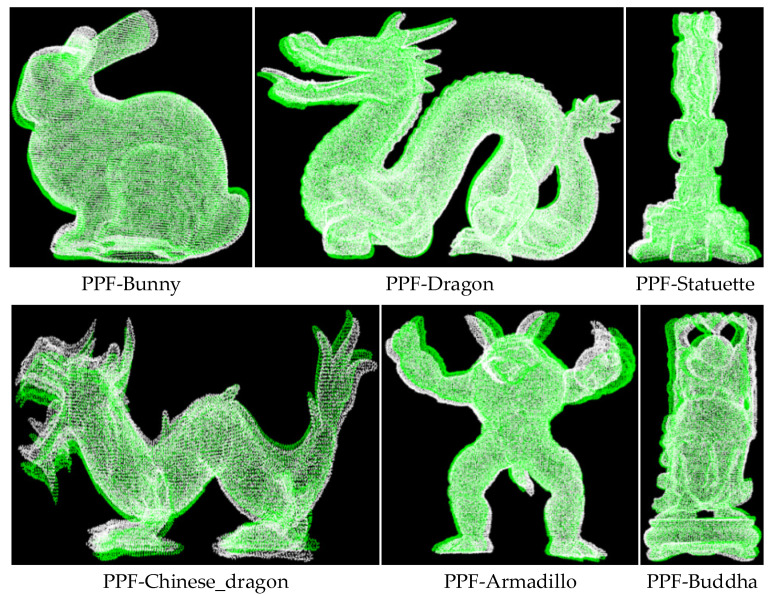
The PPF algorithm is used to register the six kinds of point cloud models of the data set. The pose results are used to convert the point cloud of the models into scene space, and the color is used for rendering, where white represents the point cloud of the scene, and green represents the converted model point cloud.

**Figure 11:**
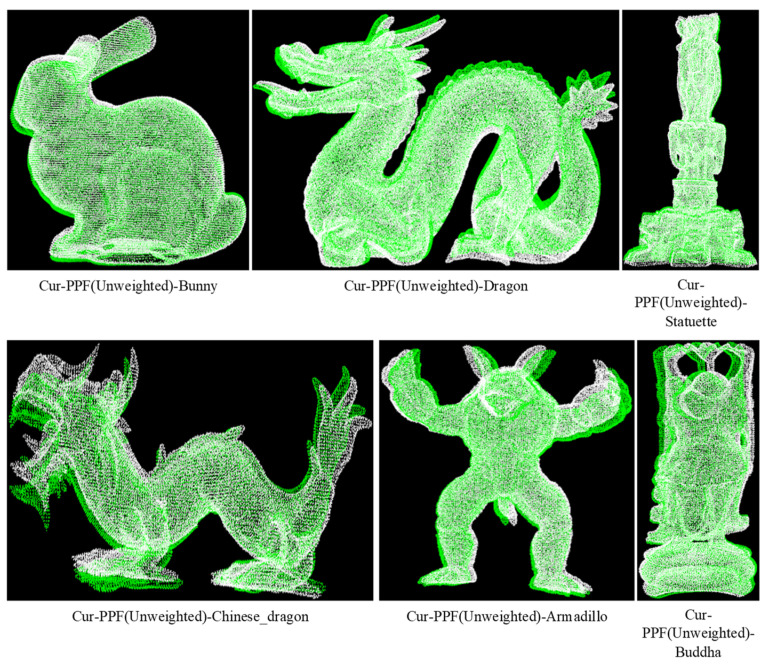
The Cur-PPF(Unweighted) algorithm is used to register the six kinds of point cloud models of the data set. The pose results are used to convert the point cloud of the models into scene space, and the color is used for rendering, where white represents the point cloud of the scene, and green represents the converted model point cloud.

We verified the enhancement effect of curvature on the PPF description in the proposed method. Each model in the Retrieval data set corresponds to multiple scenes with different levels of noise. In order to reduce the impact of noise on the matching effect, a scene with a noise coefficient of 0.1 was selected for matching. The final matching rate is the average one between each model and multiple scenes, and the average of matching time with multiple scenes is viewed as the final time. The radius of curvature of models in the data set was set to 15 mm. Due to the different curvature distributions of each model, the curvature steps $d_{cur}$ of Bunny, Dragon, Statuette, Chinese_Dragon, Armadillo, and Buddha were set to 0.07, 0.1, 0.13, 0.15, 0.2, and 0.11, respectively. The matching experiments of the PPF algorithm and the Cur-PPF (unweighted) algorithm were carried out respectively. A set of matching effects are shown in Figure 10 and Figure 11. Table 1 and Table 2 are the data comparison between the PPF algorithm and the Cur-PPF (unweighted) algorithm in terms of matching rate and time. The experimental results show that the introduction of curvature information can strengthen the description of the feature, and it is better than the original PPF algorithm in terms of matching rate and time.

**Figure 12:**
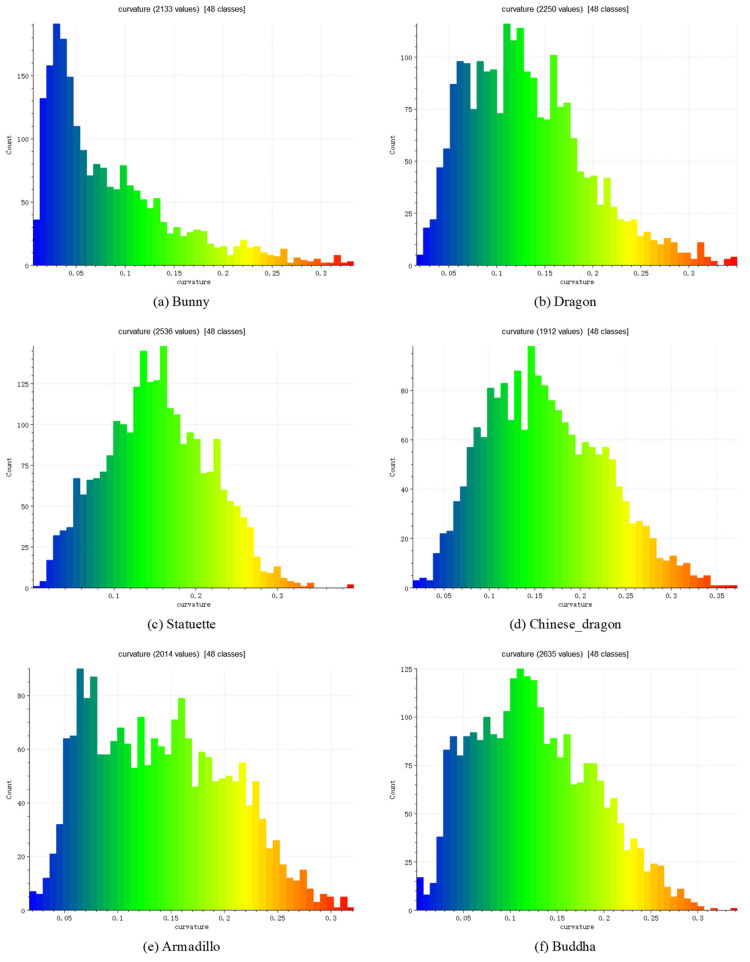
Curvature histograms of six models. The curvature of each model is divided into high/low part according to curvature histograms. (**a**) is the curvature histogram of Bunny model. Its high-curvature part is greater than 0.2 and the low-curvature part is 0–0.02; (**b**) is the curvature histogram of the Dragon model. Its high-curvature part is greater than 0.22 and the low-curvature part is 0–0.07; (**c**) is the curvature histogram of the Statuette model. Its high-curvature part is greater than 0.2 and the low-curvature part is 0–0.1; (**d**) is the curvature histogram of the Chinese_dragon model. Its high-curvature part is greater than 0.24 and the low-curvature part is 0–0.1; (**e**) is the curvature histogram of Armadillo model. Its high-curvature part is greater than 0.18 and the low-curvature part is 0–0.07; (**f**) is the curvature histogram of Buddha model. Its high-curvature part is greater than 0.18 and the low-curvature part is 0–0.07.

**Figure 13:**
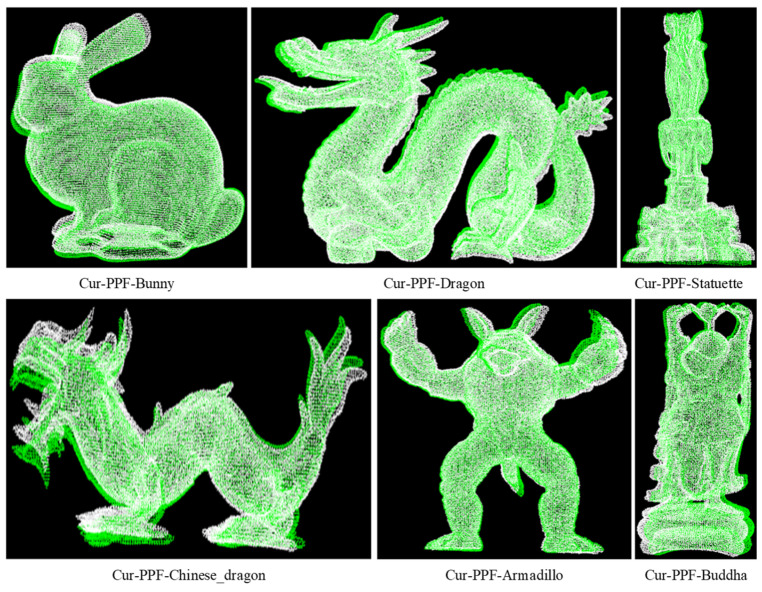
The weighted Cur-PPF algorithm is used to register the six kinds of point cloud models of the data set. The pose results are used to convert the point cloud of the models into scene space, and the color is used for rendering, where white represents the point cloud of the scene, and green represents the converted model point cloud.

We also verified that the weighted voting in the proposed method has an enhanced effect on the matching effect. According to curvature histograms of point cloud models, the high-curvature part and the low-curvature part of models are divided [35]. The curvature histograms of the models point cloud are shown in Figure 12. Through multiple experiments with different models, we think that setting the weight to 2–8 is a better range. The setting of the experimental parameters is consistent with the Cur-PPF(Unweight) parameters. The matching experiments of the Cur-PPF(unweight) algorithm and the weighted Cur-PPF algorithm were carried out respectively. The matching effect of a group of the weighted Cur-PPF algorithm are shown in Figure 13. Table 3 and Table 4 are the comparison of the matching rate and time between the Cur-PPF(Unweight) algorithm and the Cur-PPF algorithm. The experimental results show that the weighted operation introduced into the pose voting link further improve the point cloud matching rate, and the time is basically similar to the unweighted Cur-PPF algorithm, which proves the role of the weighted operation.

**Figure 14:**
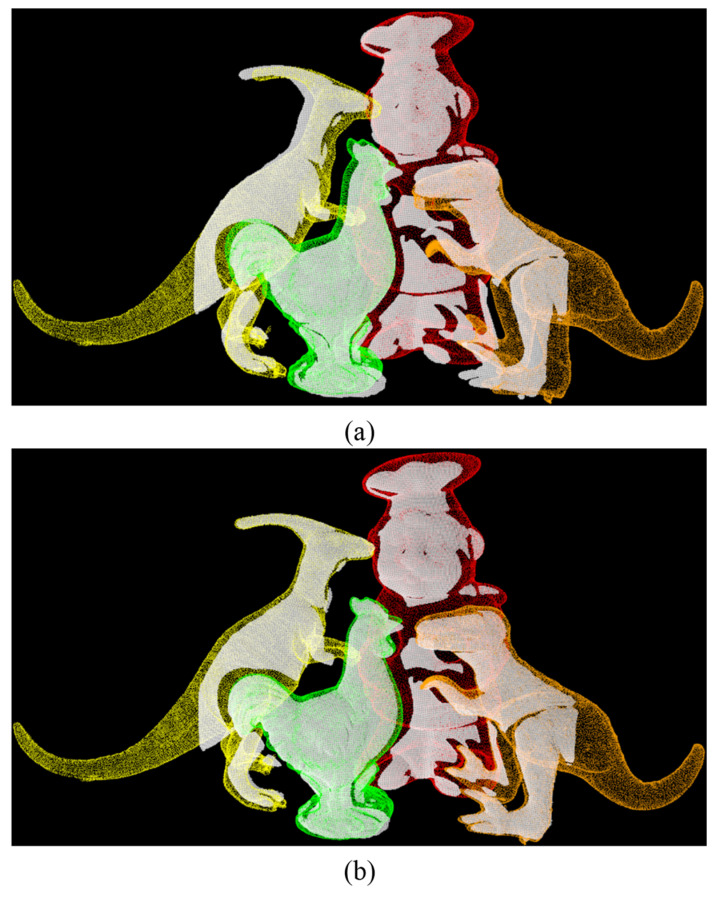
(**a**) is the result of using our proposed Cur-PPF algorithm to recognize different objects in the same scene, and (**b**) is the registration result of Figure (**a**) after optimization by the traditional ICP algorithm.

The method proposed by Drost et al. can recognize different objects in the same scene. In order to verify that the improved method proposed in this paper based on the original PPF can also effectively recognize different objects in the same scene, we choose the public dataset Laser Scanner as an experiment. Since the method in this paper focuses more on the scene of the same object in bin-picking, this experiment serves as a supplementary experiment to verify the ability of the proposed method to recognize different objects. We compared the matching rates of Cur-PPF and Cur-PPF+ICP. The results are shown in Figure 14, and the average matching rates are shown in Table 5. Experiments show that the improved method proposed in this paper has similar functions to the original PPF method, not only can identify different objects in the same scene, but also has a satisfactory coarse registration effect. After ICP optimization, the average matching rate of fine registration can reach 93%.

### 3.2. Real Scene Data

**Figure 15:**
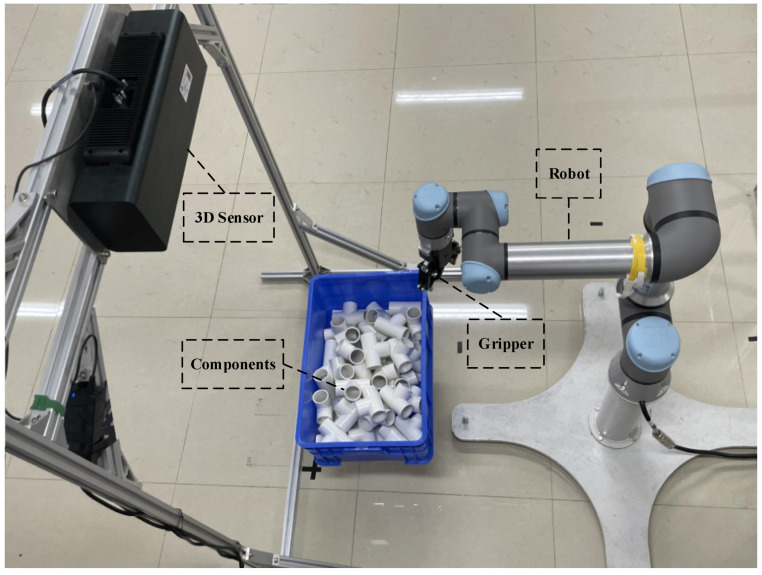
Bin-picking system diagram. The system is composed of robot, gripper, components, and 3D sensor.

In the previous section, the advantages of the proposed method Cur-PPF without clutter, overlapping occlusion are verified. However, in real scenes, the environment is chaotic and noisy, and it becomes more difficult for the robot to perform grasping tasks. In order to verify that the proposed method also has advantages in complex scenes, we built a robotic arm bin-picking scene, and the system is shown in Figure 15. The bin-picking scene is also one of the common scenes in the industry. In this scene, there is overlap and occlusion among target objects, which cause interference to the matching. In order to evaluate the algorithm, we consider the point cloud matching effect and the grasping rate of the robotic arm.

#### 3.2.1. Matching Effect of Real Scenario

In the real scenario matching experiment, we used common objects in the industry as test objects. The point cloud and image data were acquired by a 3D sensor (a COBOT COMATRIX-IM camera, consisting of a gray-scale camera and a projector). We randomly put test objects into the box, and collected 20 sets of test scenarios for each type of object, and used the PPF algorithm and the algorithm proposed in this paper to perform match experiments. The experimental parameters were set as follows: the Leaf_size of the model point cloud and scene point cloud downsampling were set to 3 mm; the hash table distance step $d_{dist}$ was set to 0.5 mm; the angle step $d_{angle}$ was set to $12^{\circ }$; the 1/5 of the point cloud number was used as the scene reference point; the radius of curvature was set to 10 mm, the curvature step $d_{cur}$ of the first type of object was set to 0.025, and the curvature step $d_{cur}$ of the second type of object was set to 0.3; the low-curvature range of the first type of object is 0–0.015, the high-curvature range is greater than 0.06, and the voting weight was set to 3; the second type of object has low-curvature range of 0–0.015, high-curvature range of greater than 0.065, and voting weight was set to 5. In calculating the matching rate between the model point cloud and the scene point cloud, the distance threshold was set to 5 mm.

**Figure 16:**
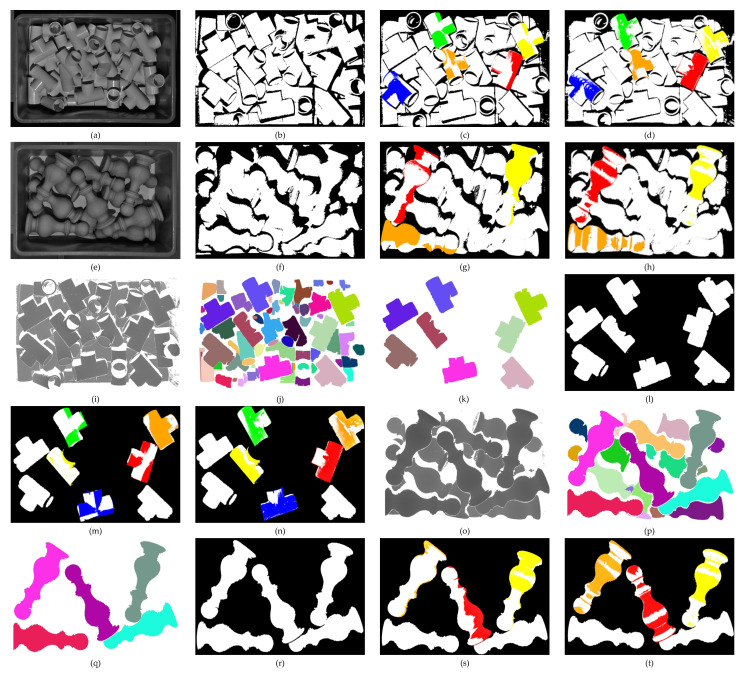
The matching process of the PPF algorithm and the Cur-PPF algorithm for two common objects in the industry. Among them, (**a**) is the three-way tube (the first type of objects); (**e**) is the upright column (the second type of objects); (**b**,**f**) correspond to the scene point cloud of two types of objects, the outer frame of the box is filtered out by setting thresholds on x−axis, y−axis respectively; (**c**,**g**) are matching results of the PPF algorithm for two types of objects; (**d**,**h**) are matching effect pictures after ICP correction; (**i**,**o**) are mapped from point cloud depth information to grayscale images; (**j**,**p**) are grayscale images after segmentation; (**k**,**q**) are candidate objects that are screened out according to the number of pixels in the segmented image; (**l**,**r**) are point clouds of candidate objects; (**m**,**s**) are the point cloud matching effect diagrams of the Cur-PPF algorithm; and (**n**,**t**) are matching effect pictures after ICP correction. The matching rate from high to low is rendered in the order of red, orange, yellow, green, and blue.

We used the PPF algorithm [17] and the Cur-PPF algorithm proposed in this paper to perform point cloud matching respectively, and the ICP algorithm was used to correct the matching results. The point cloud matching processes are shown in Figure 16. In order to effectively compare the two algorithms, we only keep the top five matching results in the scene for the first type of object. For the second type of objects, the volume of the objects is larger, and the top layer can only be placed at most five, so only the results of the top three match rates in the scene are retained. The matching results are rendered in different colors, and the average of the matching rate is regarded as the final matching rate. Table 6 and Table 7 show the comparison of the parameters of the two algorithms in terms of matching rate and time. It can be seen that the method proposed in this paper has greater advantages than the original method in the bin-picking scenario.

#### 3.2.2. Bin-Picking Performance of Robotic Arm

In order to verify the validity of the method proposed in this article, we used a six-axis robotic arm to perform bin-picking. In this system, the model of the robotic arm is UR5e (UNIVERSAL ROBOTS), the model of the gripper is AG-95 (DH ROBOTS), and the model of the 3D sensor is COMATRIX-IM (COBOT). Our experiment was carried out indoors. The light source is indoor incandescent lamp, and no specific light source is added.

We randomly placed 25 three-way tubes in the bin, and used the Cur-PPF algorithm to match the model with the scene. Each three-way tube in the scene will generate a set of pose results after weighted voting. The pose results after clustering were corrected using the ICP algorithm. According to our experience, when the matching rate is greater than 85%, the robotic arm can successfully grasp the target object. If the matching rate is less than 85%, the robotic arm will grab empty or pose error when grasping, which is considered as a wrong matching result. We carried out a total of 100 three-way tube grasping experiments, and the results showed five grasping failures, as shown in Table 8. Three of the failures were due to the close proximity of the three-way tubes, and the nearby objects were encountered before grasping, which caused the pose of the target object to change. Because of the low matching rate, which made the posture accuracy of the points captured by the robotic arm poor, and eventually led to other failure of the grasping operation.

## 4. Conclusions

We propose a 6D pose estimation method based on a new point-pair feature descriptor. In this method, an effective point cloud preprocessing is introduced, which can accurately extract candidate target objects and improve the matching efficiency. At the same time, the curvature information is introduced into the point-pair feature descriptor, which enhances the feature description and improves the matching accuracy. In addition, a weighted voting method is proposed in the pose voting link, which further improves the accuracy of pose estimation. At the end of this paper, we test the proposed method and the PPF on public data set and real scenarios. The experimental results show that the average matching rate of our method on the public data set has increased by 8.55%, and the average time taken has been shortened by 467.34 ms. In real scenarios, the average matching rate of our method has increased by 12.7%, and the average time taken has been shortened by 3188 ms, and the capture rate in the bin-picking scenarios is as high as 95%. It can be seen that the method proposed in this paper has the advantages of high pose estimation accuracy and short calculation time, and can be used in actual industrial scenarios.

In the future, we will continue to study the mathematical model of high-curvature and low-curvature partitioning in the weighting strategy, which will improve the efficiency of the strategy when applied to new objects. The point cloud matching rate can also be improved by accurately dividing the model curvature; in addition, there are useless model point-pairs during matching, and it is worth exploring how to avoid useless point-pairs in the future, which will further improve the overall efficiency.

## Figures and Tables

**Table 1:** The matching rate of PPF and Cur-PPF(Unweight) algorithms on public data set.

Models	Bunny	Dragon	Statuette	Chinese_Dragon	Armadillo	Buddha	Average
PPF [16]	87.42%	84.71%	84.92%	94.77%	81.40%	93.25%	87.75%
Cur-PPF(Unweight)	93.12%	95.96%	89.91%	95.74%	92.94%	94.25%	93.65%

**Table 2:** Time using of PPF and Cur-PPF(Unweight) algorithms on public data set (ms/scene).

Models	Bunny	Dragon	Statuette	Chinese_Dragon	Armadillo	Buddha	Average
PPF [16]	145	745	1151	893	341	803	679.67
Cur-PPF(Unweight)	85	165	169	233	203	221	179.33

**Table 3:** The matching rate of Cur-PPF(Unweight) and Cur-PPF algorithms on public data set.

Models	Bunny	Dragon	Statuette	Chinese_Dragon	Armadillo	Buddha	Average
Cur-PPF(Unweight)	93.12%	95.96%	89.91%	95.74%	92.94%	94.25%	93.65%
Cur-PPF	94.40%	99.84%	95.44%	97.09%	94.20%	96.80%	96.30%

**Table 4:** Time using of Cur-PPF(Unweight) and Cur-PPF algorithms on public data set (ms/scene).

Models	Bunny	Dragon	Statuette	Chinese_Dragon	Armadillo	Buddha	Average
Cur-PPF(Unweight)	85	165	169	233	203	221	179.33
Cur-PPF	87	195	289	226	241	236	212..33

**Table 5:** The matching rate of Cur-PPF and Cur-PPF+ICP algorithms on Laser Scanner.

Models	Cheff	Chicken	T-Rex	Parasaurolophus	Average
Cur-PPF	91.41%	87.60%	90.68%	86.01%	88.93%
Cur-PPF+ICP	95.15%	94.37%	92.86%	90.31%	93.17%

**Table 6:** The matching rate of PPF and Cur-PPF algorithms on real data sets.

Models	Three-Way Tube	Pillar	Average
PPF	83.15%	87.84%	85.50%
Cur-PPF	95.60%	94.35%	94.98%
PPF+ICP	96.10%	95.25%	95.68%
Cur-PPF+ICP	98.90%	97.50%	98.20%

**Table 7:** Time using of PPF and Cur-PPF algorithms on real data sets (ms/scene).

Models	Three-Way Tube	Pillar	Average
PPF	7034	8560	7797
Cur-PPF	3256	4236	3746
PPF+ICP	8098	9362	8730
Cur-PPF+ICP	4136	5082	4609

**Table 8:** Capture success rate for three-way tubes.

Total Number of Experiments	Success	Failure	Success Rate
100	95	5	95%

## References

[1] Inagaki Y., Araki R., Yamashita T., Fujiyoshi H. Detecting layered structures of partially occluded objects for bin picking. Proceedings of the IEEE International Conference on Intelligent Robots and Systems.

[2] Danielczuk M., Mahler J., Correa C., Goldberg K. (2018). Linear Push Policies to Increase Grasp Access for Robot Bin Picking. Proceedings of the IEEE International Conference on Automation Science and Engineering, Munich, Germany, 20–24 August 2018.

[3] Iriondo A., Lazkano E., Ansuategi A. (2021). Affordance-based grasping point detection using graph convolutional networks for industrial bin-picking applications. Sensors.

[4] Matsumura R., Harada K., Domae Y., Wan W. Learning based industrial bin-picking trained with approximate physics simulator. Proceedings of the Advances in Intelligent Systems and Computing.

[5] Hofer T., Shamsafar F., Benbarka N., Zell A. (2021). Object Detection And Autoencoder-Based 6d Pose Estimation For Highly Cluttered Bin Picking. arXiv.

[6] Chen J., Zhang L., Liu Y., Xu C. Survey on 6D Pose Estimation of Rigid Object. Proceedings of the 2020 39th Chinese Control Conference (CCC).

[7] Du G., Wang K., Lian S. (2019). Vision-based robotic grasping from object localization, pose estimation, grasp detection to motion planning: A review. arXiv.

[8] Hu Y., Hugonot J., Fua P., Salzmann M. Segmentation-Driven 6D Object Pose Estimation. Proceedings of the IEEE Computer Society Conference on Computer Vision and Pattern Recognition.

[9] Rusu R.B., Blodow N., Beetz M. Fast Point Feature Histograms (FPFH) for 3D Registration. Proceedings of the IEEE International Conference on Robotics and Automation.

[10] Tombari F., Salti S., Di Stefano L. Unique signatures of histograms for local surface description. Proceedings of the European Conference on Computer Vision.

[11] Rublee E., Rabaud V., Konolige K., Bradski G. ORB: An Efficient Alternative to SIFT or SURF. Proceedings of the IEEE International Conference on Computer Vision.

[12] Xue S., Zhang Z., Lv Q., Meng X., Tu X. (2019). Point Cloud Registration Method for Pipeline Workpieces Based on PCA and Improved ICP Algorithms. IOP Conf. Ser. Mater. Sci. Eng..

[13] Besl P.J., McKay N.D. Method for registration of 3-D shapes. Proceedings of the IEEE Transactions on Pattern Analysis and Machine Intelligence.

[14] Sarode V., Li X., Goforth H., Aoki Y., Srivatsan R.A., Lucey S., Choset H. (2019). PCRNet: Point Cloud Registration Network using PointNet Encoding. arXiv.

[15] Guo J., Xing X., Quan W., Yan D.M., Gu Q., Liu Y., Zhang X. (2021). Efficient Center Voting for Object Detection and 6D Pose Estimation in 3D Point Cloud. IEEE Trans. Image Process..

[16] Peng S., Liu Y., Huang Q., Zhou X., Bao H. PVNET: Pixel-Wise Voting Network for 6dof Pose Estimation. Proceedings of the IEEE Computer Society Conference on Computer Vision and Pattern Recognition.

[17] Drost B., Ulrich M., Navab N., Ilic S. Model globally, match locally: Efficient and robust 3D object recognition. Proceedings of the IEEE Computer Society Conference on Computer Vision and Pattern Recognition.

[18] Deng L. (2018). Artificial Intelligence in the Rising Wave of Deep Learning: The Historical Path and Future Outlook. IEEE Signal Process. Mag..

[19] Wang C., Xu D., Zhu Y., Martin-Martin R., Lu C., Fei-Fei L., Savarese S. DenseFusion: 6D object pose estimation by iterative dense fusion. Proceedings of the IEEE Computer Society Conference on Computer Vision and Pattern Recognition.

[20] Braun M., Rao Q., Wang Y., Flohr F. Pose-RCNN: Joint object detection and pose estimation using 3d object proposals. Proceedings of the IEEE Conference on Intelligent Transportation Systems.

[21] Choi C., Christensen H.I. 3D pose estimation of daily objects using an RGB-D camera. Proceedings of the IEEE International Conference on Intelligent Robots and Systems.

[22] Liu D., Arai S., Miao J., Kinugawa J., Wang Z., Kosuge K. (2018). Point pair feature-based pose estimation with multiple edge appearance models (PPF-MEAM) for robotic bin picking. Sensors.

[23] Vidal J., Lin C.Y., Lladó X., Martí R. (2018). A method for 6D pose estimation of free-form rigid objects using point pair features on range data. Sensors.

[24] Ruel S., English C., Anctil M., Church P. 3DLASSO: Real-time pose estimation from 3D data for autonomous satellite servicing. Proceedings of the Proc. ISAIRAS 2005 Conference.

[25] Mérigot Q., Ovsjanikov M., Guibas L.J. (2011). Voronoi-based curvature and feature estimation from point clouds. IEEE Trans. Vis. Comput. Graph..

[26] Beucher S., Lantuejoul C. Use of Watersheds in Contour Detection. Proceedings of the International Workshop on Image Processing.

[27] Braeger S., Foroosh H. (2018). Curvature augmented deep learning for 3D object recognition. Proceedings of the 2018 25th IEEE International Conference on Image Processing (ICIP), Athens, Greece, 7–10 October 2018.

[28] Tong L., Ying X. (2018). 3D Point Cloud Initial Registration Using Surface Curvature and SURF Matching. 3D Res..

[29] Nguyen A., Le B. 3D point cloud segmentation: A survey. Proceedings of the IEEE Conference on Robotics, Automation and Mechatronics, RAM—Proceedings.

[30] Selvarasu N., Nachiappan A., Nandhitha N.M. (2010). Euclidean Distance Based Color Image Segmentation of Abnormality Detection from Pseudo Color Thermographs. Int. J. Comput. Theory Eng..

[31] Rusu R.B., Cousins S. 3D is here: Point Cloud Library (PCL). Proceedings of the IEEE International Conference on Robotics and Automation.

[32] Wang Z., Wang E., Zhu Y. (2020). Image segmentation evaluation: A survey of methods. Artif. Intell. Rev..

[33] Xiao J., Adler B., Zhang H. 3D point cloud registration based on planar surfaces. Proceedings of the IEEE International Conference on Multisensor Fusion and Integration for Intelligent Systems.

[34] Mian A., Bennamoun M., Owens R. (2010). On the repeatability and quality of keypoints for local feature-based 3D object retrieval from cluttered scenes. Int. J. Comput. Vis..

[35] Sun J., Zhang J., Zhang G. (2016). An automatic 3D point cloud registration method based on regional curvature maps. Image Vis. Comput..

